# A De Novo Designed Metalloprotein Displays Variable Thermal Stability and Binding Stoichiometry with Transition Metal Ions

**DOI:** 10.1002/cbic.202500322

**Published:** 2025-06-27

**Authors:** Britt Rooijakkers, Gaya Verhagen, Anneloes Cramer‐Blok, Ed Zuidinga, Aimee L. Boyle

**Affiliations:** ^1^ Leiden Institute of Chemistry Leiden University Einsteinweg 55 2333 CC Leiden Netherlands; ^2^ Van 't Hoff Institute for Molecular Sciences (HIMS) University of Amsterdam PO Box 94157 1090 GD Amsterdam Netherlands; ^3^ Present address: School of Chemistry University of Bristol Cantock's Close BS8 1TS Bristol UK

**Keywords:** coiled‐coil, de novo designs, Irving–Williams series, metal binding, metalloproteins

## Abstract

Metal‐binding selectivity in natural proteins is determined by multiple factors such as the protein's structure, metal concentration within cellular compartments, and the presence of metallochaperones. The in vitro selectivity of proteins for transition metal ions is largely governed by the Irving–Williams series, which states protein‐metal complex stability follows the order Co(II) < Ni(II) < Cu(II) > Zn(II). A de novo protein has been designed that folds in the presence of certain transition metal ions into a monomeric α‐helical bundle, with the least stable protein‐metal complex being formed with Cu(II). Moreover, when increasing the metal concentration of Cu(II) or Zn(II), more metal ions are incorporated into the protein accompanied by a concurrent decrease in the amount of secondary structure. One reason may be that there is a balance between stability conferred by the coordination of the metal ion(s) and stability conferred by hydrophobic packing of the α‐helical bundle. Metals may therefore adopt distorted coordination geometries, or binding of multiple ions may cause distortion of the protein backbone, leading to compromised folding of the protein scaffold, or variable thermal stabilities of the metalloprotein complexes. This protein scaffold therefore contributes to the deciphering of design rules for metal selectivity in proteins.

## Graphical abstract

A de novo designed single‐chain coiled coil protein is unfolded in apo‐state but folds up upon addition of divalent transition metal ions Co(II), Ni(II), Cu(II), and Zn(II). The resulting metal‐bound complexes exhibit distinct thermal stability and metal stoichiometry, making this protein a promising starting point for the design of fully metal‐selective de novo proteins.

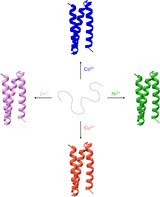

## 1 Introduction

Approximately one third of all proteins bind a metal ion and around half of all enzymes need a specific metal ion to function correctly.^[^ ^1^ ^]^ These metalloproteins play vital roles in catalysis, transport, and electron transfer.^[^ ^2^ ^]^ Although metal selectivity is of crucial importance for metalloenzymes to perform optimally, metal promiscuity can occur.^[^ ^3^ ^]^ Metalloenzymes are naturally compartmentalized and their evolved metal selectivity is largely dependent on the local concentration of metal ions within their cellular compartment. This means that, taken out of their specific environment, these enzymes often display an affinity for multiple metal ions. Due to this issue, it is often not possible to determine a relationship between metal‐binding selectivity and protein structure.^[^ ^4^ ^]^ One way this complexity can be eliminated is through studying simplified models of metal‐binding proteins created through de novo protein design.^[^ ^5^ ^]^

De novo design focuses on creating new‐to‐nature protein sequences from first principles.^[^ ^6^ ^]^ Natural proteins only sample a small section of protein sequence space (there are 20 ^200^ possible sequences for a protein of 200 residues) whereas de novo designed proteins do not necessarily adhere to this natural sequence space. Generating random sequences and seeing if they fold is arduous and time consuming, so the focus lies in designing proteins based on sequence‐to‐structure relationships.^[^ ^5^ ^]^ The goal is to design minimal models that have a similar function to natural proteins but without the structural complexity sometimes referred to as “evolutionary baggage.” In doing so it becomes easier to study and understand the direct link between a protein's structure and its function.

Many designed metalloproteins are established by incorporating metal‐binding residues into the hydrophobic core of coiled‐coil scaffolds.^[^ ^2^, ^7^ ^]^ The coiled‐coil scaffold is commonly used as it is parametrizable and sequence‐to‐structure relationships are well‐known.^[^ ^8^ ^]^ Examples of such de novo designed metalloproteins are derivatives of the α3D and DFsc scaffolds created in the DeGrado lab and utilized by both the DeGrado and Pecoraro labs.^[^ ^9^, ^10^, ^11^, ^12^ ^]^ The goal of these designs is either to create a scaffold that can later be made functional^[^ ^13^ ^]^ or to mimic the function of a natural enzyme.^[^ ^14^, ^15^, ^16^ ^]^ In more recent years, many other coiled‐coil‐based metalloproteins have been generated.^[^ ^17^, ^18^, ^19^ ^]^

Metal selectivity in de novo designed proteins is relatively unexplored. Previous studies focusing on this phenomenon incorporated metal‐binding sites between monomers of 4‐helix bundles, meaning the metal ions mediated higher‐order interactions.^[^ ^20^, ^21^ ^]^

In this article, we designed two metalloproteins, dubbed 4Hep and 3Hep. 3Hep is unfolded in the apo‐state but folds upon the addition of selected transition metal ions. The design of 3Hep is based on HisAD,^[^ ^7^ ^]^ a metallopeptide which folds and forms a trimeric coiled coil upon the addition of Cu(II) and Ni(II), but not Zn(II) and Co(II). We discovered that metal selectivity, as observed with HisAD, is not preserved in terms of folding for 3Hep. However, there is a significant difference in terms of thermal stability for all four metal ions that induced folding of 3Hep. In addition, 3Hep binds varying numbers of transition metal ions, indicating that it behaves differently for each of the metal ions examined.

## 2 Results and Discussion

### 2.1 Protein Design and Production

**Figure 1:**
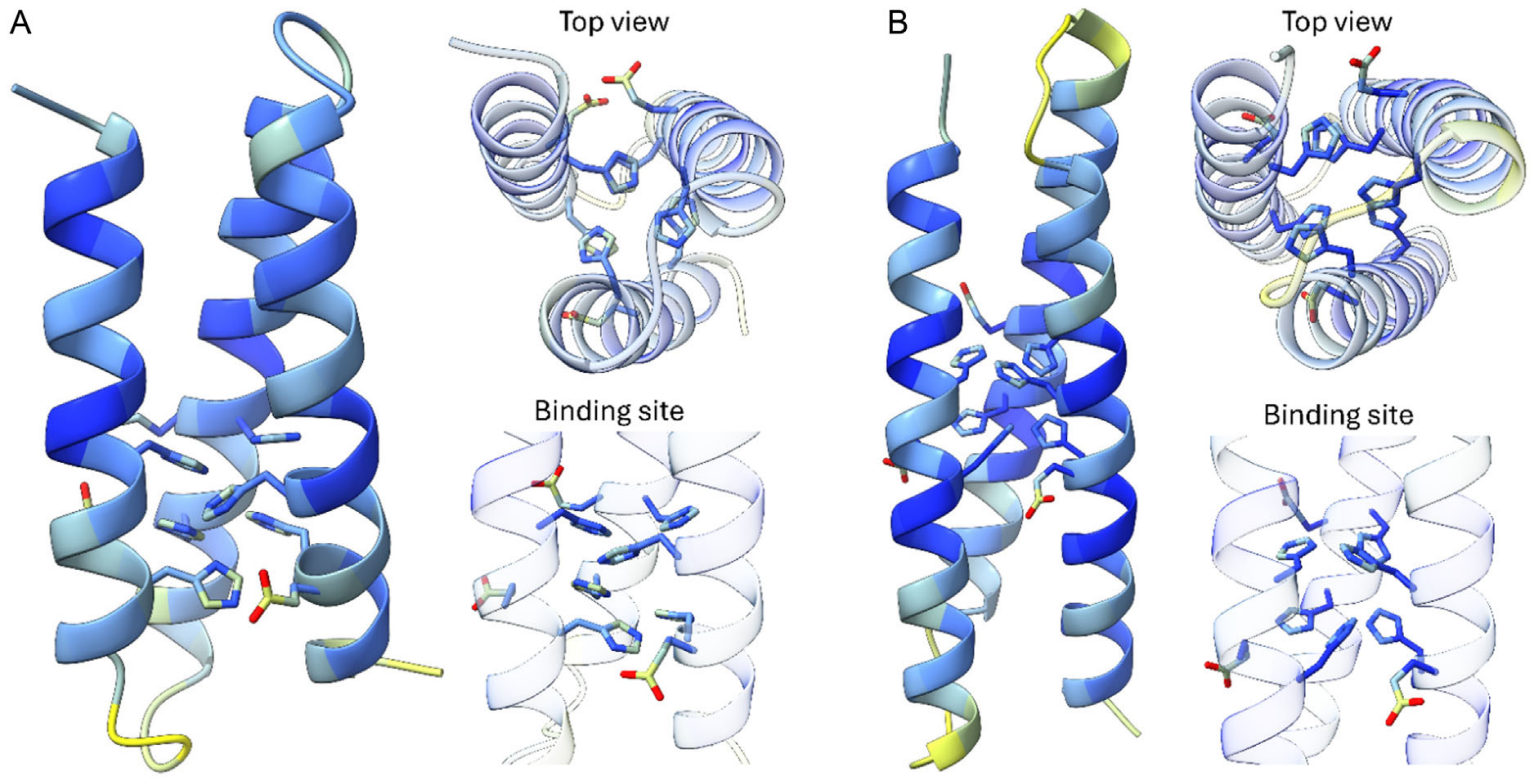
AlphaFold3^[^ ^34^ ^]^ models of A) 3Hep and B) 4Hep colored by pLDTT score. The six histidine and three glutamic acid residues designed to make up the metal‐binding site (see Table S1, Supporting Information) are shown as sticks. For the top view and the zoomed‐in view of the binding site, the cartoon representation of the helices and loops is made transparent.

We have designed two single‐chain proteins, 4Hep and 3Hep (Table S1, Supporting Information, **Figure** **1**), which were based on the selective, metal‐binding peptide HisAD.^[^ ^7^ ^]^ HisAD contained two histidine residues in each helix. The crystal structure of the HisAD:Cu(II) complex revealed a trimeric peptide complex where three Cu(II) ions were coordinated by histidine and glutamic acid residues. Therefore, we incorporated three HxxEH motifs (where *x* is either lysine, glutamic acid, or glutamine), into our 3Hep and 4Hep designs, to mimic the binding site in HisAD. 3Hep was truncated, containing only three heptad repeats per helix. This means that for 4Hep, the metal‐binding site is located in the middle of the structure, while for 3Hep the metal‐coordinating residues are closer to the C‐terminal end of the protein. AlphaFold3 (AF3) models of 3Hep and 4Hep predicted folded structures in the apo state (Figure 1), with the six histidine residues oriented toward the interior of the hydrophobic core. It is of note that the AF3 models of both proteins have similar pLDTT scores which are relatively high. This is surprising considering hydrophilic histidine residues have been incorporated into the hydrophobic core, and no metals were included in these predictions. When a metal was included in the predictions, the pLDTT scores remained similar but the PAE scores increased in comparison to predictions without a metal ion (Figure S1, Supporting Information), indicating that the prediction was more confident in the relative position of the α‐helices in the models that included one or more metal ions.

3Hep and 4Hep were produced in *Escherichia coli* (*E. coli*) BL21 Rosetta cells and purified using a combination of heat denaturation, Ni‐NTA chromatography, and size exclusion chromatography (Figure S2 and S3, Supporting Information, see materials and methods for further information). 3Hep was produced with a yield of ≈4–8 mg L^−1^ of culture. Purification of 4Hep was more challenging and yields were low, ≈0.5 mg L^−1^ of culture. We believe this is because 4Hep is already folded in the apo state (Figure S4, Supporting Information) which likely makes the histidine residues unavailable for binding the Ni‐NTA resin. We therefore decided to purify 4Hep using a strep‐tag (Table S1, Supporting Information). Yields were higher than for purification with Ni‐NTA chromatography; however, cleavage of the strep‐tag was problematic. Therefore, experiments with 4Hep were performed with the affinity tag still attached (4HepStrep, Figure S5A, Supporting Information).

### 2.2 Secondary Structure Characterization

**Figure 2:**
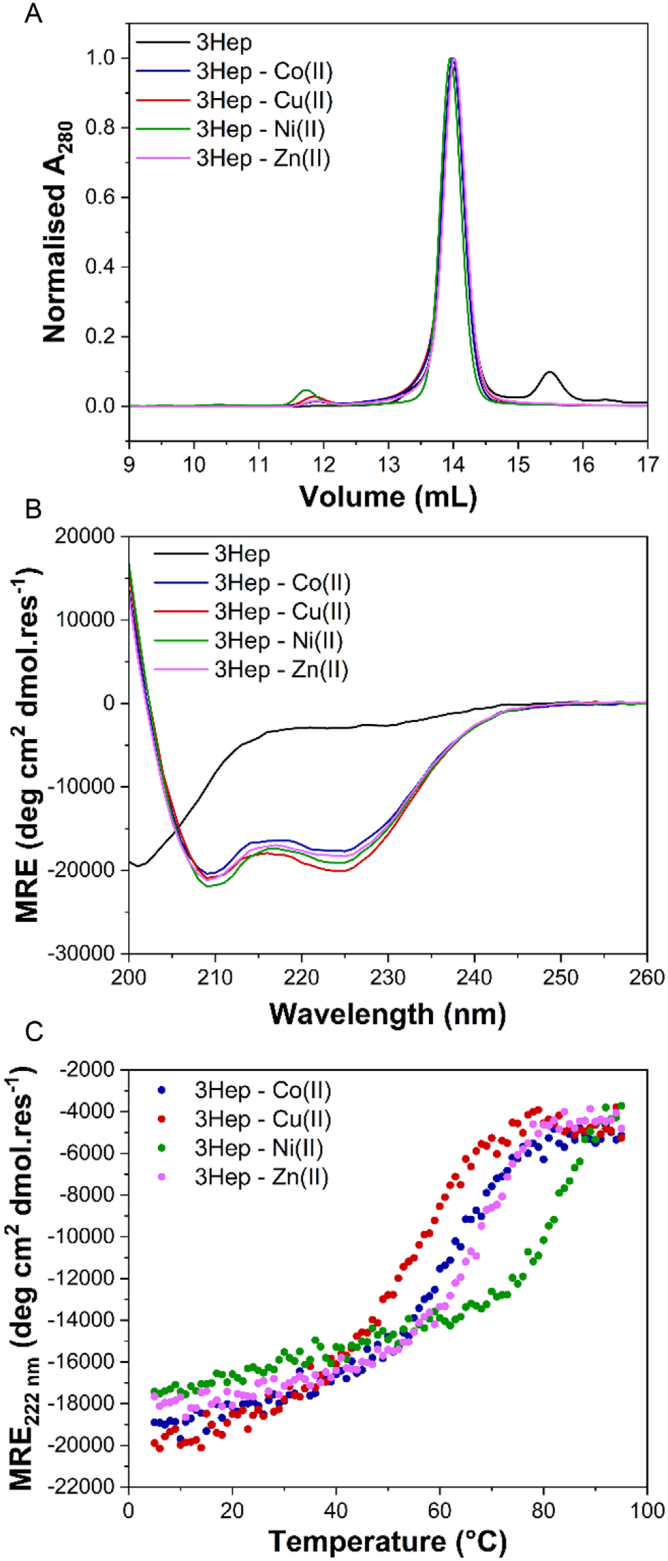
Characterization of 3Hep in the absence and presence of Co(II) (blue), Cu(II) (red), Ni(II) (green), and Zn(II) (purple). A) SEC‐MALS analysis with 100 μM 3Hep and 300 μM metal in 1*x* PBS. B) CD spectra at 20 °C and C) CD thermal melt curves of 20 μM 3Hep and 60 μM metal in 10 mM phosphate, 150 mM NaCl, pH 7.4.

Oligomeric state analysis by size exclusion chromatography‐multiangle light scattering (SEC‐MALS) showed that 3Hep is predominantly monomeric both in the apo state and in presence of either Co(II), Cu(II), Ni(II), or Zn(II) (**Figure** **2**A), as one single peak was observed and the calculated mass of the peak was equal to the mass of the monomeric protein (**Table** **1**). 4HepStrep, however, was present in a mix of oligomeric states both in the apo state and when complexed with metals (Figure S5C, Table S2, Supporting Information). Interestingly, 4HepStrep in the apo state seems to be present as a monomer and dimer in equal amounts, whereas there is a shift to the dimer state when 4HepStrep is complexed with either Co(II), Cu(II), Ni(II), or Zn(II), indicating that the dimer state is stabilized by metal binding. The 4HepStrep dimer prediction with AF3 shows a domain‐swapped dimer (Figure S5B), although the pLDTT and PAE scores are low.

**Table 1:** Secondary structure data for 3Hep in the apo state and complexed with transition metal ions in a 1:3 protein to metal ratio. Mass of the monomeric protein peak was calculated using SEC‐MALS. 3Hep's theoretical molecular weight is 7580 Da.

	Mass of the monomeric peak [kDa]	MRE_222_ [deg cm^2^ dmol.res^−1^]	*T*_m_ [°C]
Apo	7.7 ± 0.0	−2936	N.D.
Co(II)	7.7 ± 0.6	−17564	61 ± 1
Cu(II)	7.3 ± 0.1	−19721	52 ± 1
Ni(II)	7.8 ± 0.2	−18816	N.D.
Zn(II)	7.4 ± 0.2	−18160	66 ± 1

The secondary structure of 3Hep and 4HepStrep was characterized utilizing circular dichroism (CD) spectroscopy, in the absence and presence of Co(II), Cu(II), Ni(II), and Zn(II) (Figure 2B and S5D, Supporting Information). 4HepStrep was predominantly α‐helical in the apo state and no change in the CD spectrum was observed when any of the metals were added. This indicated that 4HepStrep was folded without metals. These findings, combined with the fact that 4HepStrep was not solely monomeric, led us to focus on the 3Hep construct.

Like HisAD,^[^ ^7^ ^]^ the CD spectrum indicated that 3Hep is unfolded in the apo state (Figure 2B), despite the AF3 prediction showing a folded protein. This discrepancy may be because AF3 is trained on structures from the PDB and there are structures of helical proteins that contain histidine residues and are folded,^[^ ^11^, ^22^, ^23^ ^]^ which may lead to this incorrect prediction. Folding is induced only in the presence of Co(II), Cu(II), Ni(II), and Zn(II), but not Mn(II) or Fe(II) (Figure S6, Supporting Information). This unfolding‐to‐folding transition in the presence of selected transition metal ions is of note because many de novo designed metalloproteins have pre‐organized structures.^[^ ^10^, ^11^, ^12^, ^13^, ^14^, ^15^, ^16^ ^]^ There is however some literature precedent for proteins with helices ≈3 heptads in length that contain polar residues in the hydrophobic core being unfolded.^[^ ^24^, ^25^ ^]^

There are only small differences in α‐helicity for 3Hep in complex with the different transition metal ions that induce folding. This is in contrast to HisAD, which only folded upon the addition of Cu(II) and Ni(II) and showed large differences in the amount of α‐helicity induced. We believe this behavior is likely due to entropy and folding cooperativity as the tendency for a single‐chain protein to fold around a metal ion is higher than for three separate peptides, which need to both fold and self‐assemble.

To probe the perceived loss of metal selectivity within 3Hep, thermal melt experiments were conducted and monitored via CD. These revealed a significant difference in terms of thermal stability for 3Hep complexed with the different metal ions (Figure 2C, Table 1). The most thermally stable complex was formed with Ni(II); the *T*_m_ could not be determined as the unfolding transition was not complete at 95 °C. 3Hep‐Co(II) and 3Hep‐Zn(II) have similar stabilities, with *T*_m_s of 61 ± 1 °C and 66 ± 1 °C respectively. 3Hep‐Cu(II) was the least stable with a *T*_m_ of 52 ± 1 °C. This order of thermal stability was unexpected. We hypothesize that the lower thermal stability of the 3Hep‐Cu(II) complex relative to the other complexes could be due to the fact that the 3Hep structure is more constrained than HisAD and so Cu(II) may not be able to bind in a preferred coordination geometry; this is observed in other de novo designed proteins,^[^ ^21^ ^]^ and in Nature with, for example, the NikR transcription factor.^[^ ^26^, ^27^, ^28^, ^29^ ^]^

### 2.3 Metal Stoichiometry

**Figure 3:**
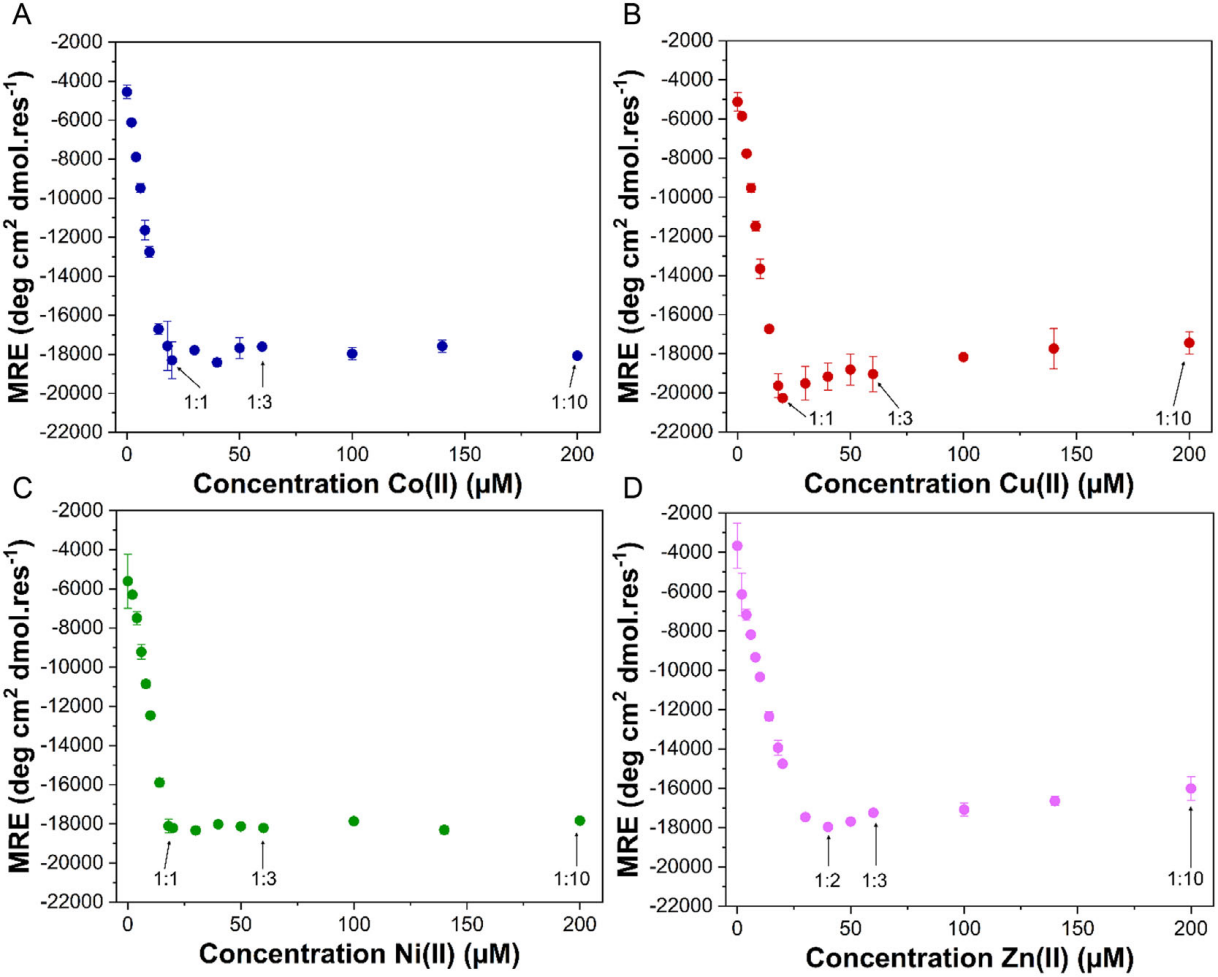
CD titrations of 3Hep with transition metals A) Co(II), B) Cu(II), C) Ni(II), and D) Zn(II) at 20 °C with 20 μM 3Hep and increasing amounts of metal in 10 mM phosphate, 150 mM NaCl, pH 7.4. The MRE at 222 nm is plotted against the concentration of metal. Protein to metal ratios are indicated for some titration points. Data points represent the mean of two independent measurements. Error bars indicate the standard deviation of two repeats.

**Figure 4:**
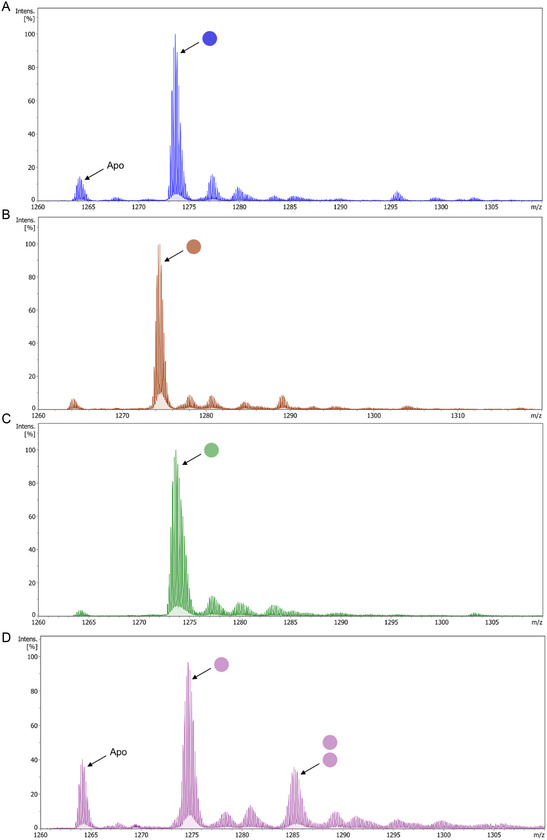
Native electrospray ionization mass spectrometry of 3Hep with transition metals A) Co(II) at 1:1, B) Cu(II) at 1:1, C) Ni(II) at 1:1, and D) Zn(II) at 1:2 protein‐to‐metal ratios in 20 mM ammonium acetate. Only the 6+ peaks are shown. Samples contained 15 μM 3Hep and 15 μM metal for the 1:1 spectra and 30 μM metal for the 1:2 spectrum. Peaks that represent 3Hep in the apo state are indicated, and 3Hep complexed with metal ions are indicated by arrows with circles representing the number of metal ions bound.

Metal titrations monitored using CD spectroscopy were performed to investigate stoichiometry of binding. For Co(II), Cu(II) and Ni(II) saturation of the α‐helical fold was reached at a 1:1 protein:metal ratio, while for Zn(II) this was at a 1:2 protein:metal ratio (**Figure** **3**). For Co(II) and Ni(II), the α‐helicity does not change after the saturation point, indicating that these metals bind to 3Hep in a 1:1 stoichiometry. Interestingly, for Cu(II) and Zn(II), the α‐helicity is decreased after the saturation point, which could indicate a change in the number of metal ions binding, potentially accompanied by an alteration of coordination geometry, which affects the folding of 3Hep. We attempted to fit these data; however, for Cu(II) and Zn(II) in particular, this was not straightforward. Therefore, we turned to native mass spectrometry to gain more insight as to the metal stoichiometry. Native mass spectrometry experiments were conducted with 3Hep in the apo state (Figure S12, Supporting Information) and in the presence of Co(II), Cu(II), Ni(II), or Zn(II) at their respective saturation points as determined from the CD titrations (1:2 for Zn(II) and 1:1 for the other metals) and also in a 1:1 (for Zn(II)), 1:3, and 1:10 protein‐to‐metal ratio (**Figure** **4** and S13–S16, Supporting Information). We chose to measure these ratios to study the effect of an increased metal concentration; as for Cu(II) and Zn(II), this proved to have an effect on the overall fold.

3Hep complexed with Ni(II), the most stable complex out of all the metals tested, shows one clear peak representing 3Hep + 1 Ni(II) ion (Figure 4 and S15, Supporting Information) at all protein‐to‐metal ratios. At 1:10, a small peak appears representing 3Hep + 2 Ni(II) ions. 3Hep‐Co(II) shows the same trend (Figure 4A and S13, Supporting Information). This is in line with the CD titration data, as no change in helicity is observed for either 3Hep‐Co(II) or 3Hep‐Ni(II) at protein:metal ratios higher than 1:1. Therefore, we decided to fit a simple 1‐ion binding model to the CD titration data by calculating the fraction folded^[^ ^30^ ^]^ (Figure S8 and S9, Supporting Information, materials and methods); however, binding was too tight to calculate accurate *K*_D_ values. By simulating the curves at different *K*_D_ values, we were able to determine that the *K*_D_ values for 3Hep + 1 Ni(II) and 3Hep + 1 Co(II) are either equal to or lower than 10 nM.

The measured mass spectra for 3Hep‐Cu(II) and 3Hep‐Zn(II) do show a shift going from the 1:1, 1:2, to 1:3 and 1:10 protein:metal ratios (Figure 4B and S13, Supporting Information, and Figure 4D and S15, Supporting Information, respectively). 3Hep‐Cu(II) at 1:1 and 1:3 ratios shows one peak representing the 3Hep + 1 Cu(II) species, while at the 1:10 ratio there are two peaks for 3Hep + 1 Cu(II) and 3Hep + 2 Cu(II) species, together with a smaller peak representing 3Hep + 3 Cu(II). Based on the CD titration data, we assume that the incorporation of more Cu(II) ions into the 3Hep protein is disruptive to the overall fold since the α‐helicity of the complex decreases when increasing the metal concentration. We assume that which exact species are formed and how many Cu(II) ions are bound are likely the result of a delicate balance between stability conferred by coordination of Cu(II) into its preferred geometry and distortion of the protein backbone from its preferred helical configuration, resulting in the disruption of α‐helical secondary structure.

For Zn(II), at a 1:2 protein:metal ratio, there is a peak representing 3Hep + 1 Zn(II) and a smaller peak representing 3Hep + 2 Zn(II). The peak representing the apo species is also clearly present, indicating a lower affinity for this metal ion, which can also be inferred from the CD titration data. The spectrum at the 1:1 ratio also shows a peak for 3Hep + 2 Zn(II), indicating that even when metal ions are not in excess, the formation of a 2 ion species is favorable. When increasing the protein:metal ratio to 1:3 and 1:10, the peak representing 3Hep + 2 Zn(II) dominates and a peak representing 3Hep + 3 Zn(II) appears. This is interesting because the same did not happen for Cu(II), indicating that for Zn(II) the 2‐ion species might be more favored than the 1‐ion species, in line with the titration data. In addition, the coordination numbers for metals Co(II), Cu(II), Ni(II), and Zn(II) commonly found in protein structures in the Protein Data Bank (PDB) are 6 for Co(II) and Ni(II) and 4 for Cu(II) and Zn(II),^[^ ^31^, ^32^ ^]^ which could explain why for Cu(II) and Zn(II) more metal ions are bound to 3Hep at higher protein‐to‐metal ratios. We tried to fit both a 1:1 and 1:2 binding model to the CD titration data for Cu(II) and Zn(II) (Figure S10 and S11, Supporting Information), and it is clear that the 1‐ion binding model does not fit the data well and the 1:2 binding model fits better. However, the native MS experiments reveal a mix of 1‐ion and 2‐ion species, in addition to a 3‐ion species for Zn(II). Therefore, this 1:2 binding model is likely not representative either and a complex equilibrium of multiple species is probably formed; therefore, K_D_'s could not be determined for Cu(II) or Zn(II).

To probe the effects that the formation of multiple ion species may have on 3Hep thermal stability, we measured additional thermal melts for 3Hep‐Cu(II) and 3Hep‐Zn(II) at 1:1 and 1:10 ratios for both metals and at a 1:2 ratio for 3Hep‐Zn(II) (Figure S7, Table S3, Supporting Information). 3Hep‐Cu(II) at a 1:1 ratio has a *T*_m_ of 57 ± 0 °C, compared to 52 ± 1 °C for 3Hep‐Cu(II) at 1:3 and 1:10 ratios. Native MS measurements at 1:1 and 1:3 ratios do not show a difference, as only the 1‐ion species peak is visible; the 2‐ion peak becomes visible at a 1:10 ratio. Interestingly, the most thermally stable ratio is also the ratio at which the protein is most α‐helical. Conversely, for 3Hep‐Zn(II), a 1:2 ratio (where the protein is most α‐helical) shows a lower *T*_m_ of 62 ± 1 °C, compared to 66 ± 1 °C at all other protein‐to‐metal ratios. It should also be noted that for 3Hep‐Zn(II), the unfolding curve for the 1:1 ratio is less sigmoidal, indicating a shift from cooperative to noncooperative unfolding. Therefore, we cannot directly correlate thermal stability to the number of metal ions bound. The thermal stability appears to be influenced by many factors: how many metal ions are bound and in what coordination geometry, whether unfolding is cooperative, and for Zn(II) at least, multiple metal ion species are present as different protein‐to‐metal ratios and therefore the unfolding curves are the average of several different species.

Based on the CD titration and native MS data, we hypothesize that the thermal stabilities for Ni(II) and Co(II) complexes, Ni(II) being more stable than Co(II), are probably a reflection of the Irving–Williams series.^[^ ^33^ ^]^ More complicated processes are occurring for Cu(II) and Zn(II) as the amount of metal ions changes when the metal concentration is increased which makes it difficult to determine metal‐binding affinity. More work on probing the coordination geometries and structures of the different protein‐metal complexes needs to be performed. Several attempts at crystallizing these complexes were made, but diffraction‐quality crystals could not be obtained. In addition, investigation of the secondary coordination sphere is necessary, as in most metalloproteins this greatly affects metal‐binding affinity and selectivity, because it can stabilize specific coordination geometries or encapsulate the metal‐binding site.^[^ ^32^ ^]^

## 3 Conclusions

We have successfully designed a metalloprotein, 3Hep, based on our previously reported metal‐selective self‐assembling peptide. 3Hep is unfolded in the apo state but folds in the presence of Co(II), Cu(II), Ni(II), and Zn(II). Thermal stability of 3Hep bound to Cu(II) was, surprisingly, the lowest for all transition metal ions tested. 3Hep bound to Ni(II) formed the most stable complex, followed by 3Hep‐Zn(II) and 3Hep‐Co(II), which had similar stabilities. The amount of metal ions bound is dependent on the metal concentration for Cu(II) and Zn(II), which showed a small change in conformation after the saturation point. For these metals, there were also multiple metal ions bound at higher metal concentrations. This was not the case for Co(II) and Ni(II), where only one metal ion‐bound species was observed even at higher metal concentrations. We assume the reason that 3Hep folds up around Co(II), Cu(II), Ni(II), and Zn(II) while HisAD does not is because the general stability and tendency of 3Hep to fold is higher due to folding cooperativity and entropy. The reason that 3Hep‐Cu(II) is the least thermally stable complex could be because Cu(II) cannot bind in its preferred coordination geometry without significantly disrupting the α‐helical secondary structure unlike, for example, Co(II) and Ni(II). More research needs to be done to probe the coordination geometry and structure of all 3Hep metal complexes in order to uncover the reasons behind this unusual thermal stability. With this information we plan to design new metalloprotein scaffolds that exclusively bind one type of metal ion, achieving full metal selectivity.

## 4 Experimental Section

4.1

4.1.1

##### Protein Production and Purification

The pET28a plasmid containing the gene for 3Hep was ordered from GeneArt (Invitrogen, Thermo Fisher Scientific) and transformed into *E. coli* BL21 Rosetta cells. Transformants were grown overnight at 37 °C on LB agar plates containing 50 μg mL^−1^ kanamycin and 34 μg mL^−1^ chloramphenicol. Starter cultures were grown overnight from single transformants in LB medium containing 50 μg mL^−1^ kanamycin and 34 μg mL^−1^ chloramphenicol. Starter cultures were diluted 1:100 in fresh LB medium containing the same antibiotic concentrations and grown at 37 °C while shaking. When the OD600 reached ≈0.6, cultures were induced with IPTG at a final concentration of 0.5 mM and cells were grown for ≈4 h at 25 °C while shaking. Cells were harvested by centrifugation at 1925 × *g*, 4 °C for 20 min. Cells were lysed using a French Press and the lysate was centrifuged at 126 087 × *g*, 4 °C for 30 min. The supernatant was heat denatured at 70 °C for 30 min and the heat denatured lysate was centrifuged at 126 087 × *g*, 4 °C for 30 min. Protein was purified first with Ni‐NTA chromatography, since the protein was unfolded and has six available histidines to bind to the Ni‐NTA resin, followed by size exclusion chromatography on a Superdex 75 column (Cytiva) using a buffer containing 10 mM phosphate, 150 mM NaCl, pH 7.4. Contents of the SEC fractions were checked by sodium dodecyl sulfate polyacrylamide gel electrophoresis (SDS‐PAGE) analysis and purity of the final protein sample was confirmed by TOF mass spectrometry (Figure S2 and S3, Supporting Information).

##### SEC‐MALS Analysis

SEC‐MALS analysis was performed using a system containing a miniDAWN TREOS, DynaPro NanoStar, Opti‐lab differential refractometer (Wyatt technology), and 1260 Infinity II multiple wavelength absorbance detector (Agilent). Samples were run on a Superdex Increase 75 10/300 column (Cytiva) using 1x PBS as the running buffer at a flow rate of 0.75 mL min^−1^. Data was processed using the ASTRA 8 software package.

##### Circular Dichroism Spectroscopy

Measurements were performed using a Jasco J‐1500 CD Spectrometer at 20 °C in a 1 mm quartz cuvette. Spectra were recorded from 260 to 190 nm with a scanning speed of 100 nm min^−1^, data pitch of 1 nm, digital integration time (D.I.T.) of 1 s, and a bandwidth of 1 nm. For each measurement, 5 scans were recorded and averaged. For the full spectrum measurements, 3 measurements were performed for freshly prepared samples and averaged.

CD signal was converted to mean residue ellipticity (MRE) using Equation (1).

(1) $$MRE=\frac{{\left[\theta \right]}_{\text{observed}}}{C\times l\times n}\times 100$$

where [*θ*]_observed_ is the observed CD signal, *C* is the concentration of protein, *l* is the path length, and *n* is the number of amino acids of the protein.

CD thermal melts were recorded from 5 to 95 °C with a temperature slope of 1 °C min^−1^. CD signal at 222 nm was recorded every 1 °C. Thermal melt curves were fit to the Boltzmann equation using Origin Pro software. *T*_m_s were determined by taking the derivative of the fit. Measurements were performed twice and *T*_m_s determined by the Boltzmann fits for each measurement were averaged. Samples for both full spectrum measurements and thermal melts contained 20 μM protein and 20, 40, 60, or 200 μM MCl_2_ (where M designates Co(II), Cu(II), Ni(II), or Zn(II)) for protein to metal ratios 1:1, 1:2, 1:3, and 1:10, respectively, in 10 mM phosphate, 150 mM NaCl, pH 7.4.

For the titration experiments, fresh samples were prepared for each titration point. Samples had a protein concentration of 20 μM and varying concentrations of MCl_2_ ranging from 0 to 200 μM in 10 mM phosphate, 150 mM NaCl, pH 7.4. CD titration curves were fit to an equation derived from a simple 1‐ion binding model, shown in Equation (2).

(2) 3Hep+1M(II)↔3HepM(II)

The dissociation constant, *K*_D_, of this equation is defined in Equation (3).

(3) $$K_{D}=\frac{\left[3\text{Hep}\right]\left[M\left(\text{II}\right)\right]}{\left[3\text{HepM}\left(\text{II}\right)\right]}$$

where [3Hep] is the concentration of 3Hep in apo state, [M(II)] is the free metal concentration, and [3HepM(II)] is the concentration of the protein‐metal complex. We have expressed this equation as the total protein concentration [3Hep]_T_ and total metal concentration [M(II)]_T_ and the fraction folded *f*, as described in Peacock, A. et al. (2014),^[^ ^30^ ^]^ shown in Equation (4) and (5).

(4) $$f=\frac{\theta_{\text{observed}}-\theta_{\text{free}}}{\theta_{\text{saturated}}-\theta_{\text{free}}}$$

(5) $$K_{D}=\frac{({\left[3\text{Hep}\right]}_{T}-f{\left[3\text{Hep}\right]}_{T})\left({\left[\text{Cu}\left(\text{II}\right)\right]}_{T}-f{\left[3\text{Hep}\right]}_{T}\right)}{f{\left[3\text{Hep}\right]}_{T}}$$

where *θ*_observed_ is the observed molar ellipticity, *θ*_saturated_ is the molar ellipticity of the protein in its most folded state, and *θ*_free_ is the molar ellipticity of the protein in the absence of metal.

This function can be solved for *f*, shown in Equation (6).

(6) $$f=\frac{\pm \sqrt{{{\text{K}}_{\text{D}}}^{2}+\text{2}{\text{K}}_{\text{D}}({\left[3\text{Hep}\right]}_{T}+{\left[\text{Cu}(\text{II})\right]}_{T})+{\left({\left[3\text{Hep}\right]}_{T}-{\left[\text{Cu}(\text{II})\right]}_{T}\right)}^{2}}}{2{\left[3\text{Hep}\right]}_{T}}+\frac{{\text{K}}_{\text{D}}+{\left[3\text{Hep}\right]}_{T}+{\left[\text{Cu}(\text{II})\right]}_{T}}{2{\left[3\text{Hep}\right]}_{T}}$$

This equation was used to fit the CD titration curves using Origin Pro software, but *K*_D_'s could not be determined because binding was too tight. Instead, simulated curves were produced using Equation (5) and the *K*_D_ was set manually to find a maximal *K*_D_ value for CD titrations with Co(II) and Ni(II) (Figure S8 and S9, Supporting Information).

CD titration curves for Cu(II) and Zn(II) were also fit to a 2‐ion binding model (Figure S10 and S11, Supporting Information), which was derived from Equation (5), shown in Equation (7).
(7)

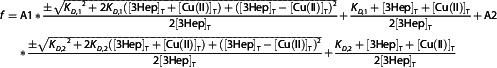

where A1 and *K*_D,1_ are the amplitude of the curve and dissociation constant for the first binding step, respectively, and *A2* and *K*_D,2_ are the amplitude of the curve and dissociation constant for the second binding step.

##### Native Mass Spectrometry

ESI‐MS experiments were performed using a HR‐ToF, Impact II of Bruker Daltonik GmbH (Bremen, Germany) with an Electro Spray Ionization source. Detection was in positive mode. The capillary voltage was between 3.5 and 4.5 kV. The source temp was held at 60 °C. The sample was infused with a syringe pump at a flow rate of 180 μl h^−1^. The machine was calibrated prior to the experiment with TFA‐Na solution, which provided a m/z range of single charged peaks up to 3000. The system was washed with EDTA and then NH_4_CH_3_CO_2_ before and in‐between measurements to ensure the system was free of metal ions. For sample preparation, a 3Hep protein stock was incubated with a 5× molar excess of EDTA for 1 h on ice before buffer exchange to 20 mM NH_4_CH_3_CO_2_. Samples were prepared with 15 μM protein with or without 45 or 150 μM MCl_2_. For each sample, 100–150 spectra were obtained during 1–2 min and averaged and processed using DataAnalysis 5.3 software. Resolution for the 6+ charge state was around 25 000.

## Conflict of Interest

The authors declare no conflict of interest.

## Supporting information

Supplementary Material

## Data Availability

The data that support the findings of this study are available from the corresponding author upon reasonable request.
