# Supplementary material for: A De Novo Designed Metalloprotein Displays Variable Thermal Stability and Binding Stoichiometry with Transition Metal Ions
Source: Chembiochem. 2025 Jun 27;26(14):e202500322. doi: 10.1002/cbic.202500322 (PMC12278340; doi:10.1002/cbic.202500322)
Supplement: Supplementary file 1 — Supplementary Material [file CBIC-26-e202500322-s001.pdf]

SUPPORTING INFORMATION

A *De Novo* Designed Metalloprotein Displays Variable Thermal Stability and Binding Stoichiometry with Transition Metal Ions

Britt Rooijakkers[a], Gaya Verhagen[a], Anneloes Cramer-Blok[a], Ed Zuidinga[c], and Aimee L. Boyle*[a] [b]

[a] B. Rooijakkers, G. Verhagen, A. Cramer-Blok, Dr A. L. Boyle
Leiden Institute of Chemistry
Leiden University
Einsteinweg 55, 2333 CC Leiden

[b] Dr. A.L. Boyle (current address)
School of Chemistry
University of Bristol
Cantock's Close
Bristol
BS8 1TS
UK
E-mail: aimee.boyle@bristol.ac.uk

[c] E. Zuidinga
Van 't Hoff Institute for Molecular Sciences (HIMS)
University of Amsterdam
PO Box 94157
1090 GD Amsterdam

Table S1. Peptide sequence of HisAD and the derived protein sequences for 4Hep and 3Hep. Sequences for 4Hep and 3Hep can be read from left to right, top to bottom. Histidine and glutamic acid residues that are likely to coordinate transition metal ions are shown in bold and underlined.

Peptide/protein			Sequence				
			gabcdef	*gabcdef*	*gabcdef*	*gabcdef*	
HisAD		G	EIAAIKQ	EIAA**H**KK	**EH**AAIKW	EIAAIKQ	GYG
			gabcdef	*gabcdef*	*gabcdef*	*gabcdef*	
4Hep		G	EIAAIKQ	EIAA**H**KK	**EH**AAIKW	EIAAIKQ	GSGG
			KIAAIKQ	KIAA**H**KQ	**EH**AAIKK	KIAAIKQ	GSGG
			EIAAIEQ	EIAA**H**EK	**EH**AAIEQ	EIAAIEQ	
			gabcdef	*gabcdef*	*gabcdef*		
3Hep		G	EIAAIKQ	EIAA**H**KK	**EH**AAIKW	GSGG	
			KIAA**H**KQ	**EH**AAIKK	KIAAIKQ	GSGG	
			EIAAIEQ	EIAA**H**EK	**EH**AAIEQ		
	Strep-tag sequence		*gabcdef*	*gabcdef*	*gabcdef*	*gabcdef*	
4HepStrep	AWSHPQFEKENLYFQ	G	EIAAIKQ	EIAA**H**KK	**EH**AAIKW	EIAAIKQ	GSGG
			KIAAIKQ	KIAA**H**KQ	**EH**AAIKK	KIAAIKQ	GSGG
			EIAAIEQ	EIAA**H**EK	**EH**AAIEQ	EIAAIEQ	

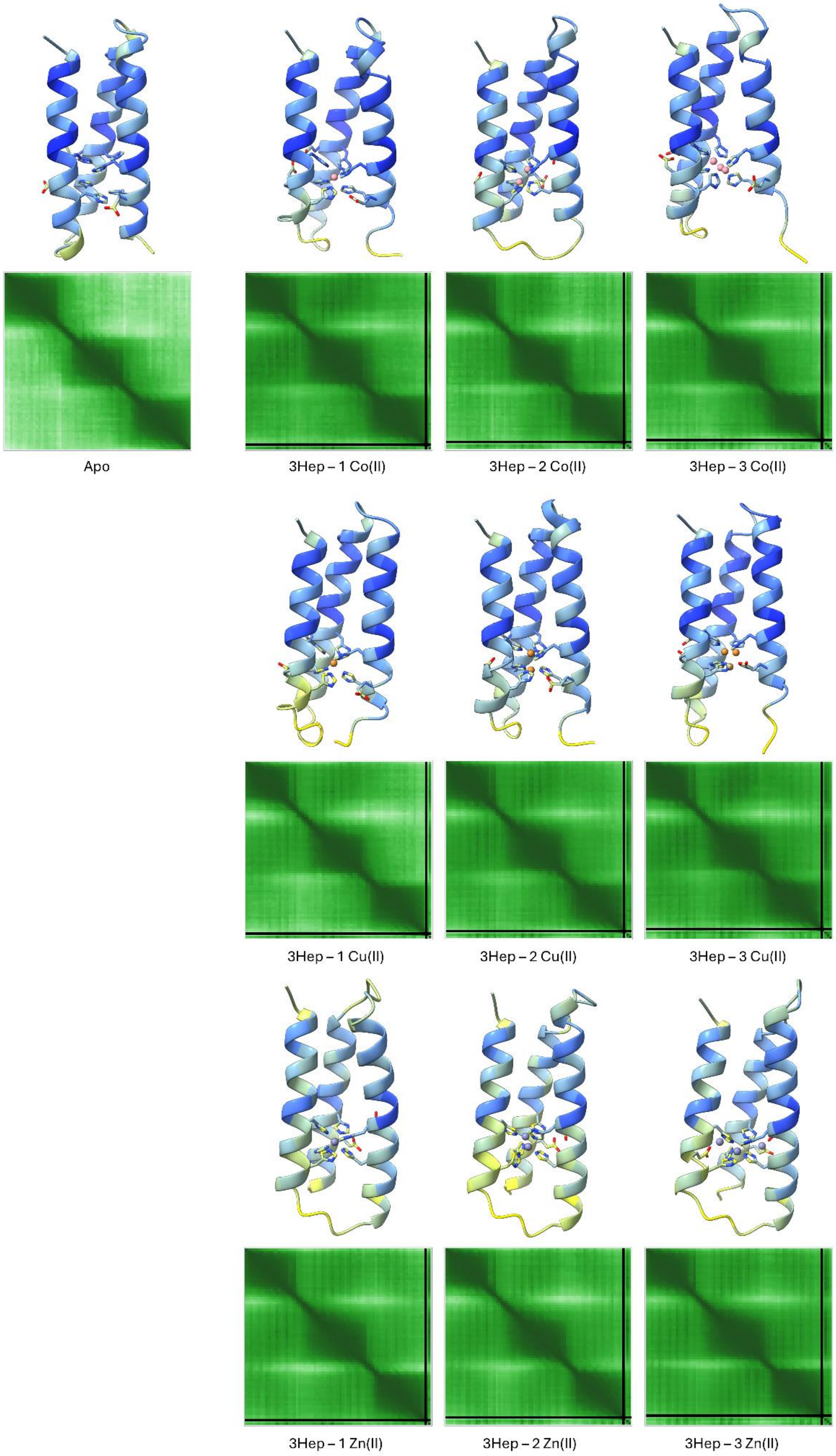

Figure S1. AlphaFold3 models and PAE plots of 3Hep in the apo state and complexed with metals, colored by pLDTT score. Histidine and glutamic acid residues designed to make up the metal-binding site (see Table S1) are shown as sticks and colored blue for nitrogen atoms and red for oxygen atoms.

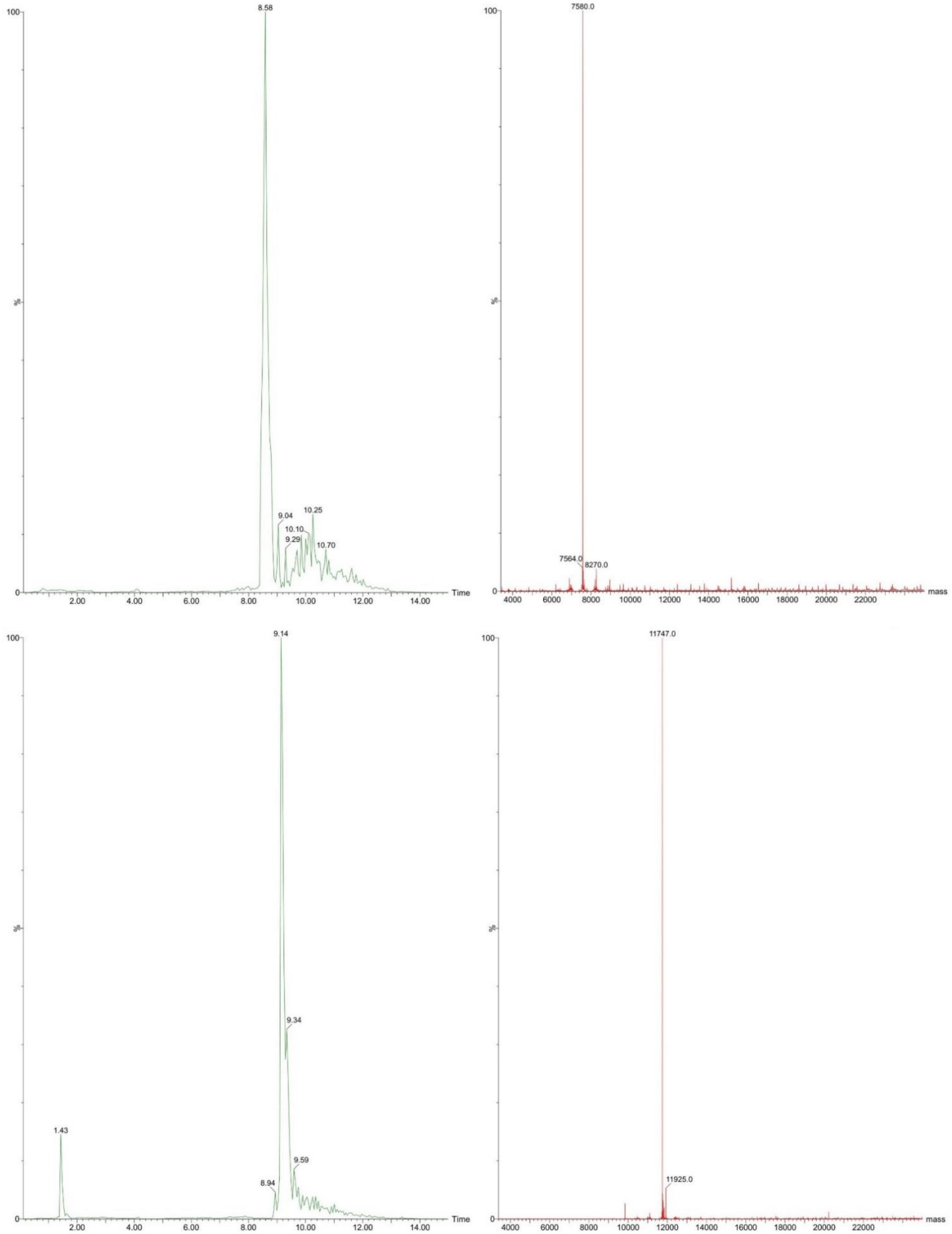

Figure S2. TOF MS spectra for 3Hep (top) and 4HepStrep (bottom). Left shows the chromatogram in green and right shows the deconvoluted spectrum in red. Mass of the peaks are indicated in Da. 3Hep theoretical mass = 7579.65 Da, 3Hep observed mass = 7580.0 Da. 4HepStrep theoretical mass = 11747.43 Da, 4HepStrep observed mass = 11747.0 Da.

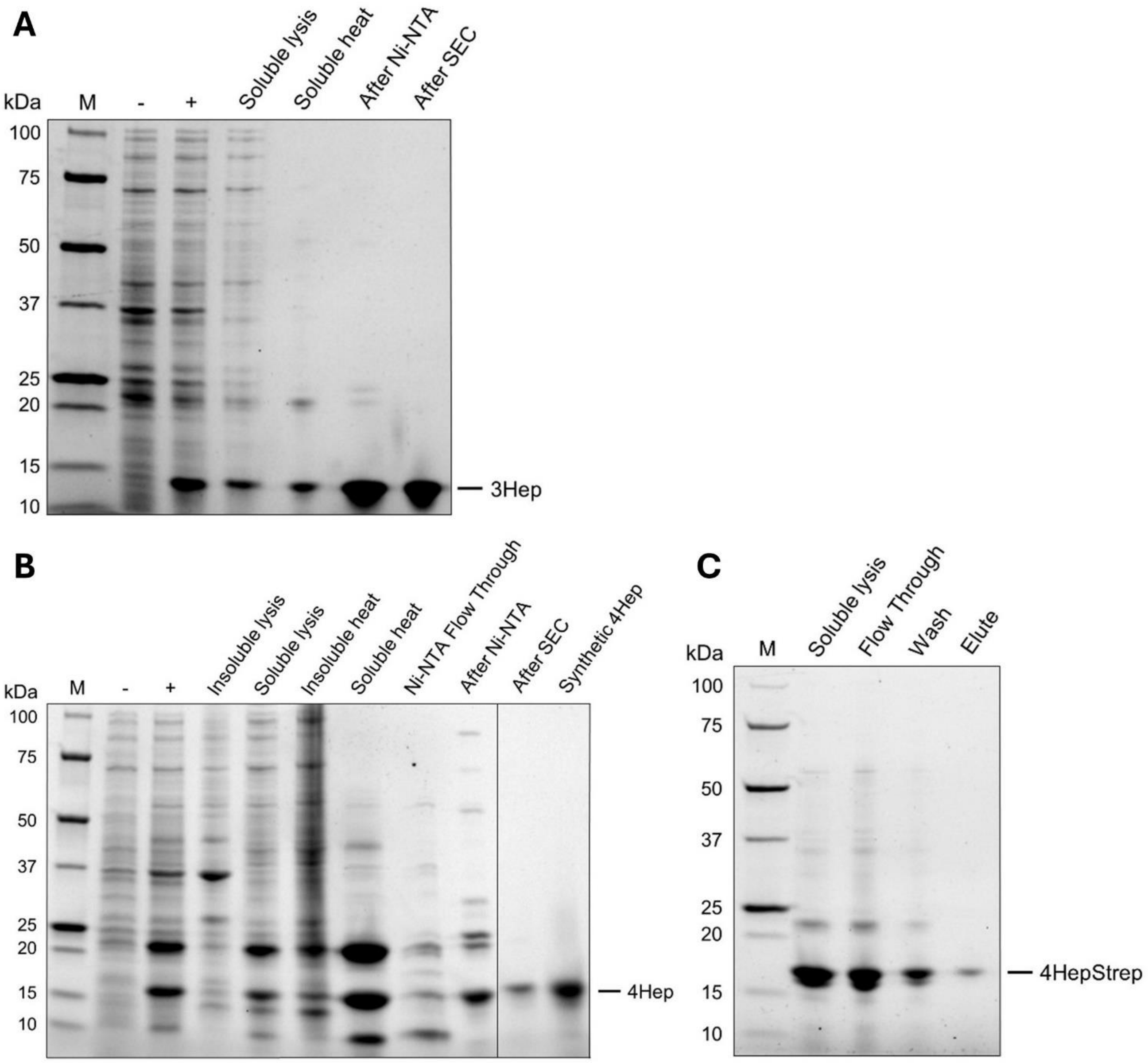

Figure S3. SDS-PAGE analysis of 3Hep (A), 4Hep (B) and 4HepStrep (C) overexpression in Rosetta DE3 cells and/or purification with heat denaturation, Ni-NTA chromatography and size exclusion chromatography. Samples were loaded on TGX 4-15% Stain-Free precast gels (Bio-Rad) and run for 45 min at 180 V.

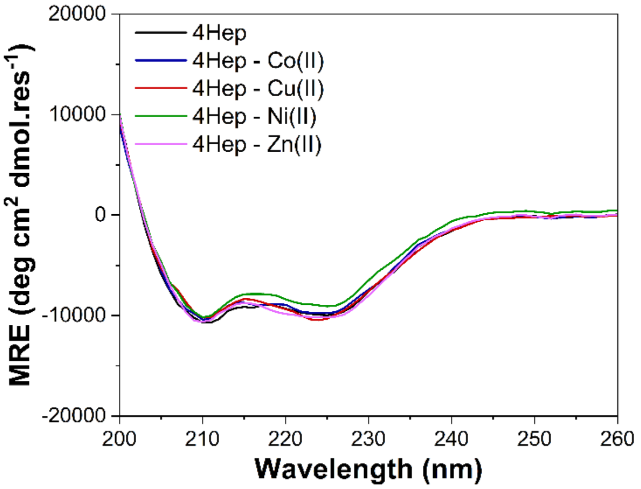

Figure S4. CD spectra at 20 °C of 4Hep in absence (black) and presence of metal ions Co(II) (blue), Cu(II) (red), Ni(II) (green) and Zn(II) (purple) in 10 mM phosphate, 100 mM NaCl, pH 7.4.

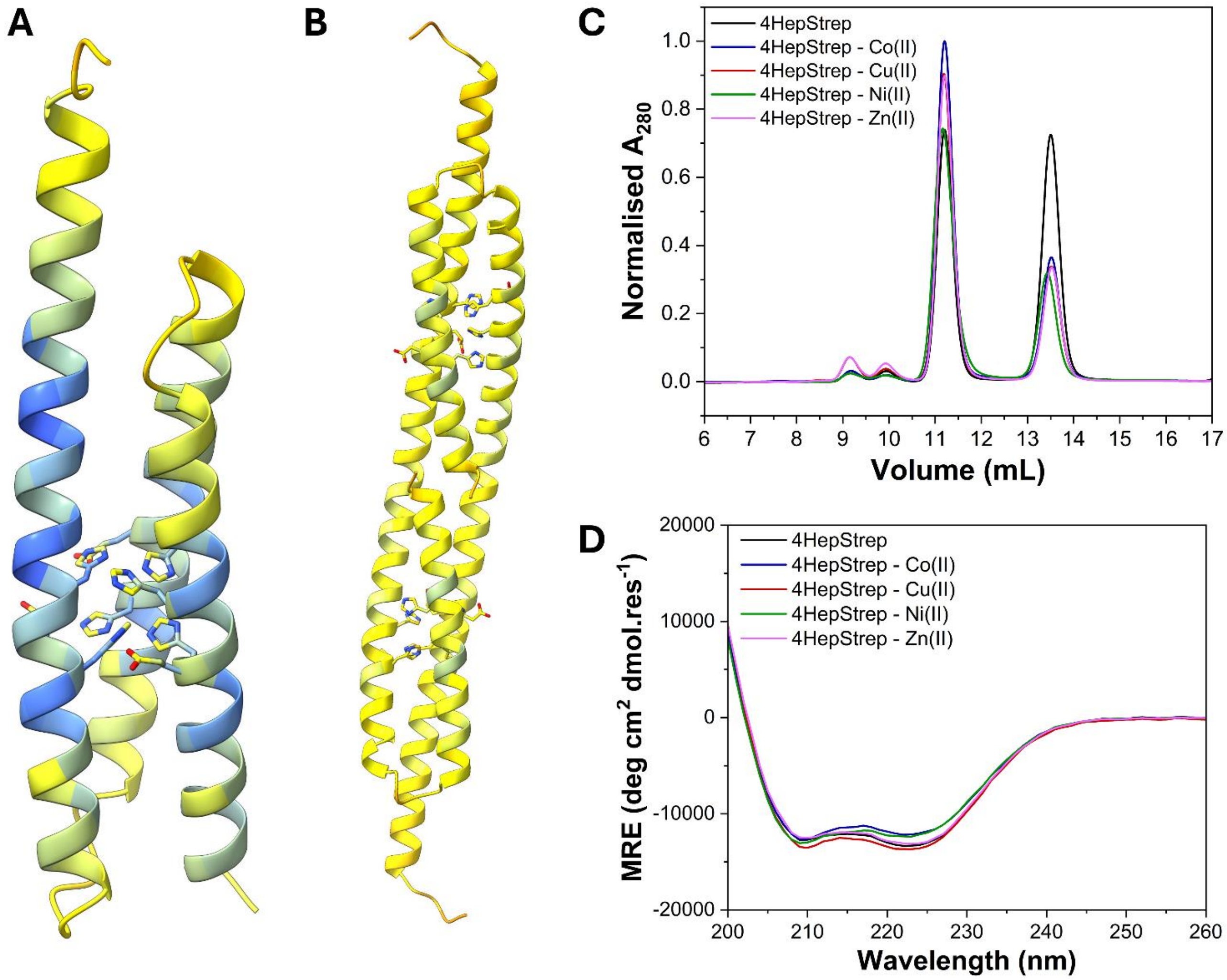

Figure S5. AlphaFold3 models and secondary structure data for 4HepStrep. **A)** AlphaFold3 model of 4HepStrep monomer and **B)** domain-swapped dimer. Models are coloured according to pLDTT values. **C)** SEC-MALS analysis of 100 μM 4HepStrep with and without 300 μM Co(II), Cu(II), Ni(II) or Zn(II) in 10 mM phosphate, 150 mM NaCl, pH 7.4. **D)** CD spectra of 20 μM 4HepStrep with and without 60 μM Co(II), Cu(II), Ni(II) or Zn(II) in 10 mM phosphate, 150 mM NaCl, pH 7.4.

Table S2. Secondary structure data for 4HepStrep in the apo state and complexed with transition metal ions. Total mass fraction of the monomer and dimer was determined by SEC-MALS. Data is averaged over two runs.

	Total mass fraction monomer (%)	Total mass fraction dimer (%)	MRE_{222} (deg cm^2 dmol.res^{-1})
Apo	49 ± 6	48 ± 8	-13332
Co(II)	32 ± 4	74 ± 4	-12178
Cu(II)	30 ± 1	68 ± 0	-13657
Ni(II)	32 ± 3	66 ± 0	-12347
Zn(II)	29 ± 0	63 ± 1	-13073

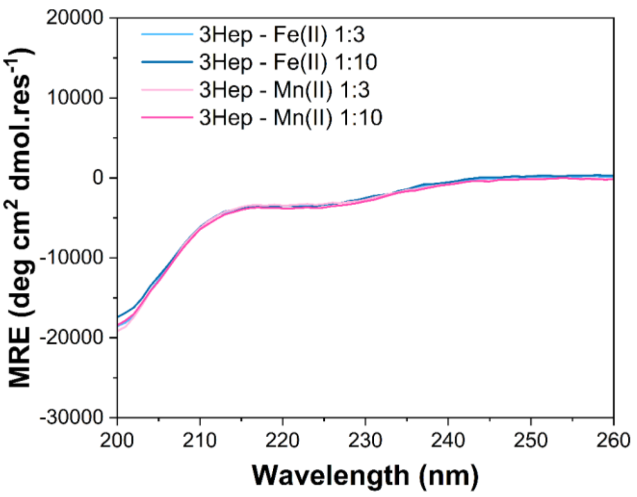

Figure S6. CD spectra of 3Hep in the presence of Mn(II) (pink) and Fe(II) (blue) at protein:metal ratios of 1:3 and 1:10. Samples contained 20 μM 3Hep and 60 μM or 200 μM $MnCl_2$ or $FeCl_2$ for the 1:3 and 1:10 measurements, respectively, in 10 mM phosphate, 150 mM NaCl, pH 7.4, 20 °C.

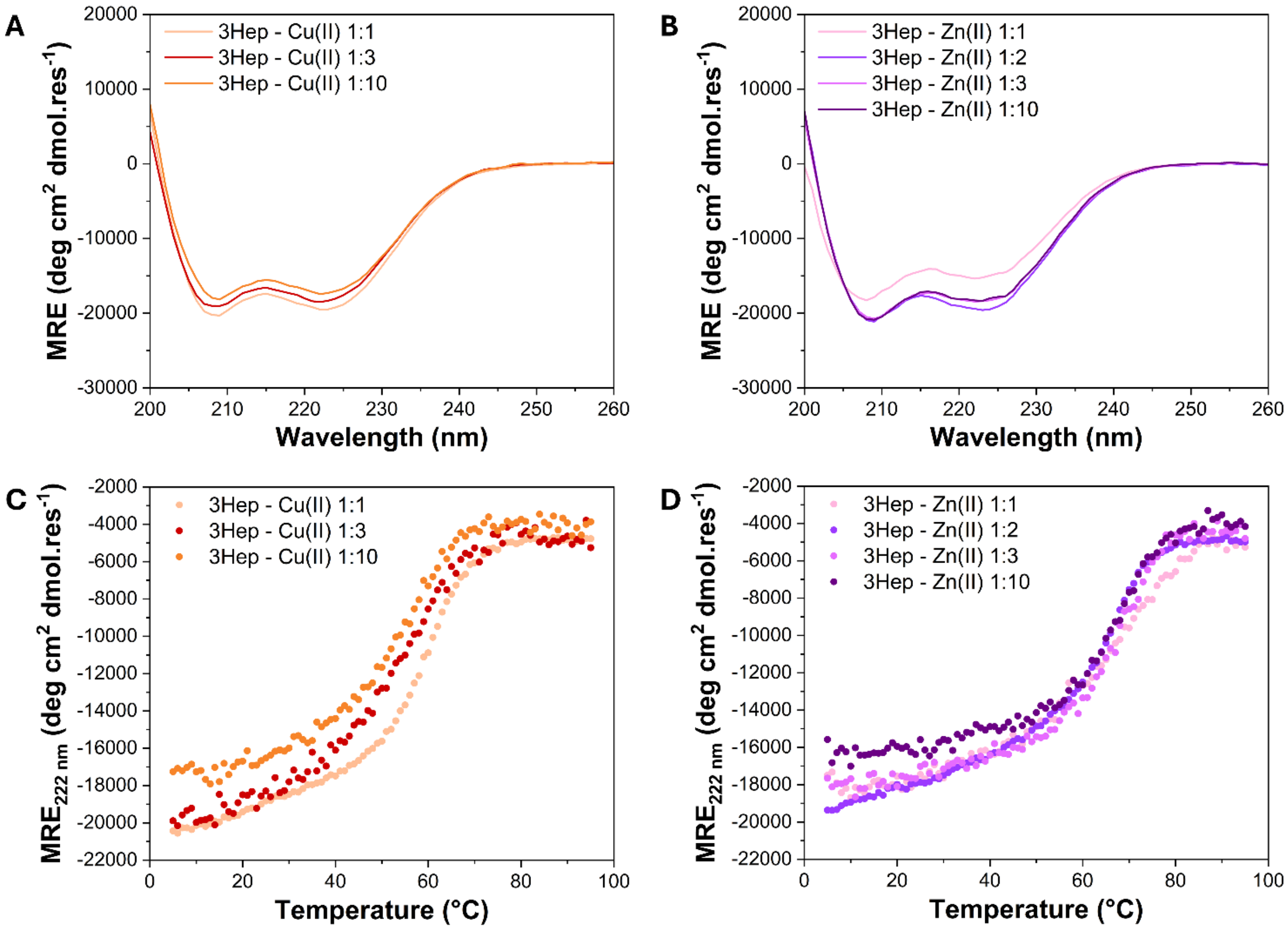

Figure S7. CD full spectra and thermal melt curves for 3Hep in the presence of Cu(II) at a 1:1, 1:3 and 1:10 metal:protein ratio (A, C) and in the presence of Zn(II) at a 1:1, 1:2, 1:3 and 1:10 metal:protein ratio (B, D). Samples contained 20 µM 3Hep and 20 µM, 40 µM, 60 µM or 200 µM metal for the 1:1, 1:2, 1:3 and 1:10 metal:protein ratios, respectively, in 10 mM phosphate, 150 mM NaCl, pH 7.4. CD full spectra were measured at 20 °C.

Table S3. Melting temperatures for 3Hep complexed with Cu(II) and Zn(II) at different protein:metal ratios, determined by CD thermal melt data.

Metal	Protein:metal ratio	T_m (°C)
Cu(II)	1:1	57 ± 0
	1:10	52 ± 1
Zn(II)	1:1	66 ± 1
	1:2	62 ± 1
	1:10	66 ± 1

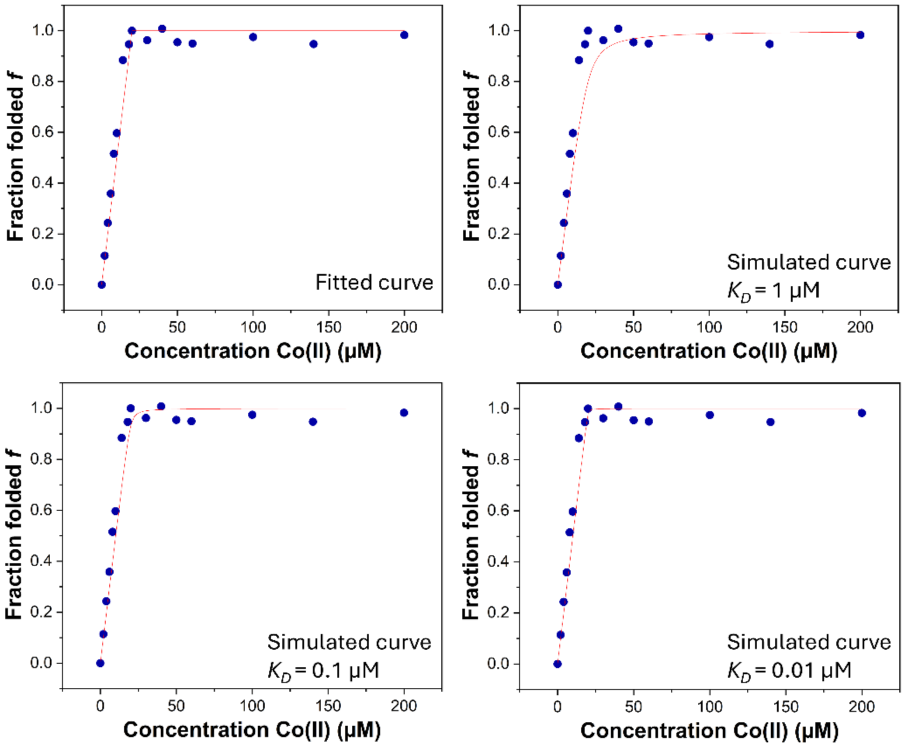

Figure S8. CD metal titrations for 3Hep (20 µM) in the presence of varying concentrations of Co(II). Fraction folded was calculated according to Equation 3. Red lines represent either best fits (top left) or simulated curves for K_D = 1 µM (top right), K_D = 0.1 µM (bottom left) or K_D = 0.01 µM (bottom right) using Equation 6.

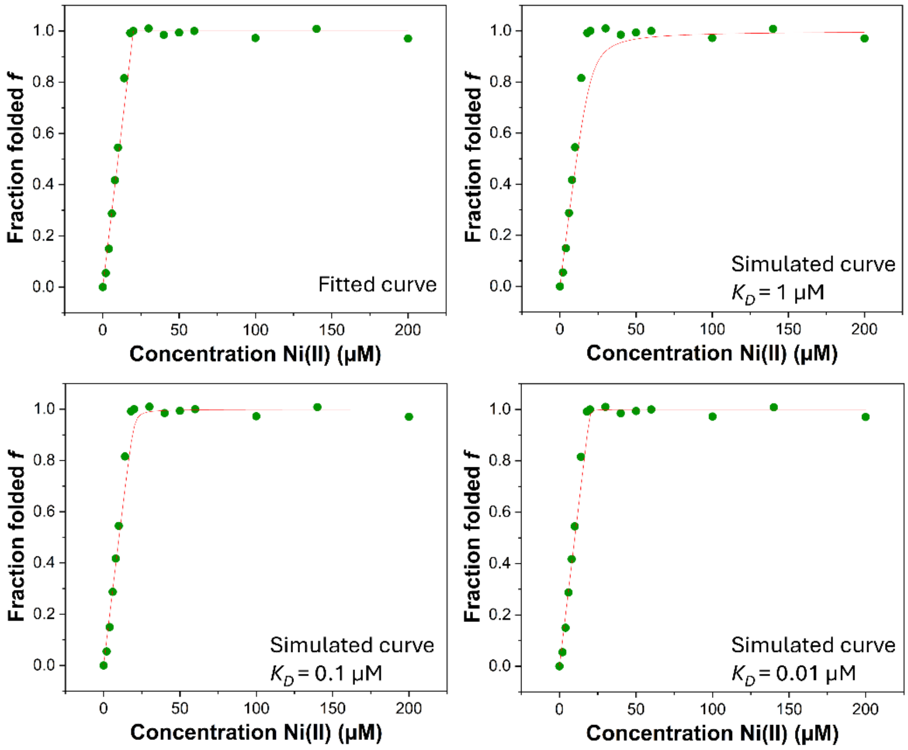

Figure S9. CD metal titrations for 3Hep (20 µM) in the presence of varying concentrations of Ni(II). Fraction folded was calculated according to Equation 3. Red lines represent either best fits (top left) or simulated curves for K_D = 1 µM (top right), K_D = 0.1 µM (bottom left) or K_D = 0.01 µM (bottom right) using Equation 6.

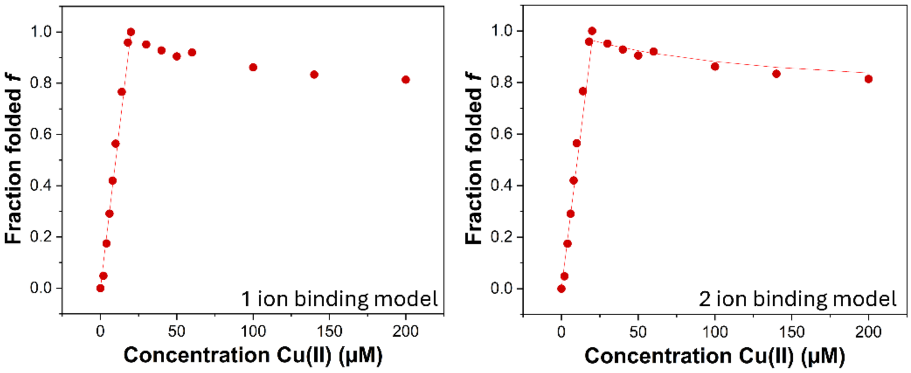

Figure S10. CD metal titrations for 3Hep (20 μM) in the presence of varying concentrations of Cu(II). Fraction folded was calculated according to Equation 3. Red lines represent either best fits using Equation 6 (for a 1-ion binding model) or Equation 7 (for a 2-ion binding model).

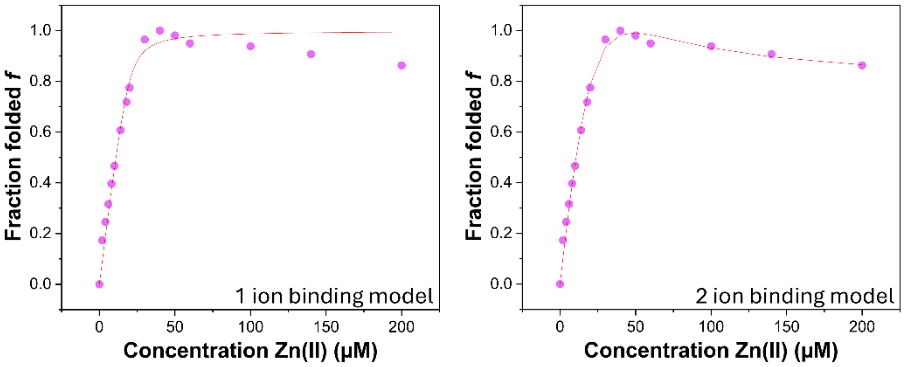

Figure S11. CD metal titrations for 3Hep (20 μM) in the presence of varying concentrations of Zn(II). Fraction folded was calculated according to Equation 3. Red lines represent either best fits using Equation 6 (for a 1-ion binding model) or Equation 7 (for a 2-ion binding model).

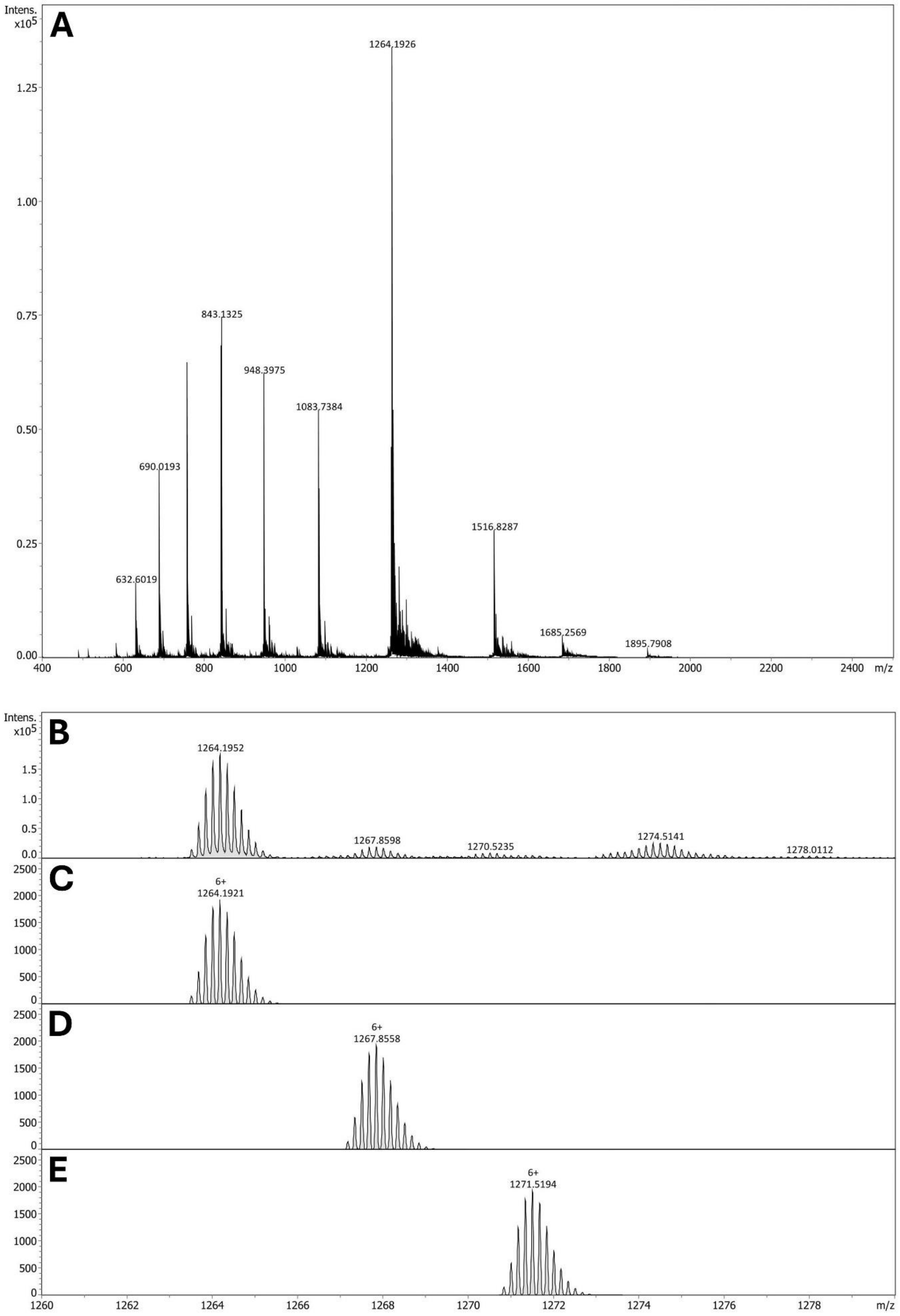

Figure S12. Native electrospray ionization mass spectrometry of 15 µM 3Hep in 20 mM ammonium acetate. **A)** Full spectrum. **B)** Measured spectrum of 3Hep. Only the 6^{+} peaks are shown. **C)** Simulated mass for 3Hep. **D)** Simulated mass for 3Hep sodium adduct with 1 Na^{+}. **E)** Simulated mass for 3Hep sodium adduct with 2 Na^{+}.

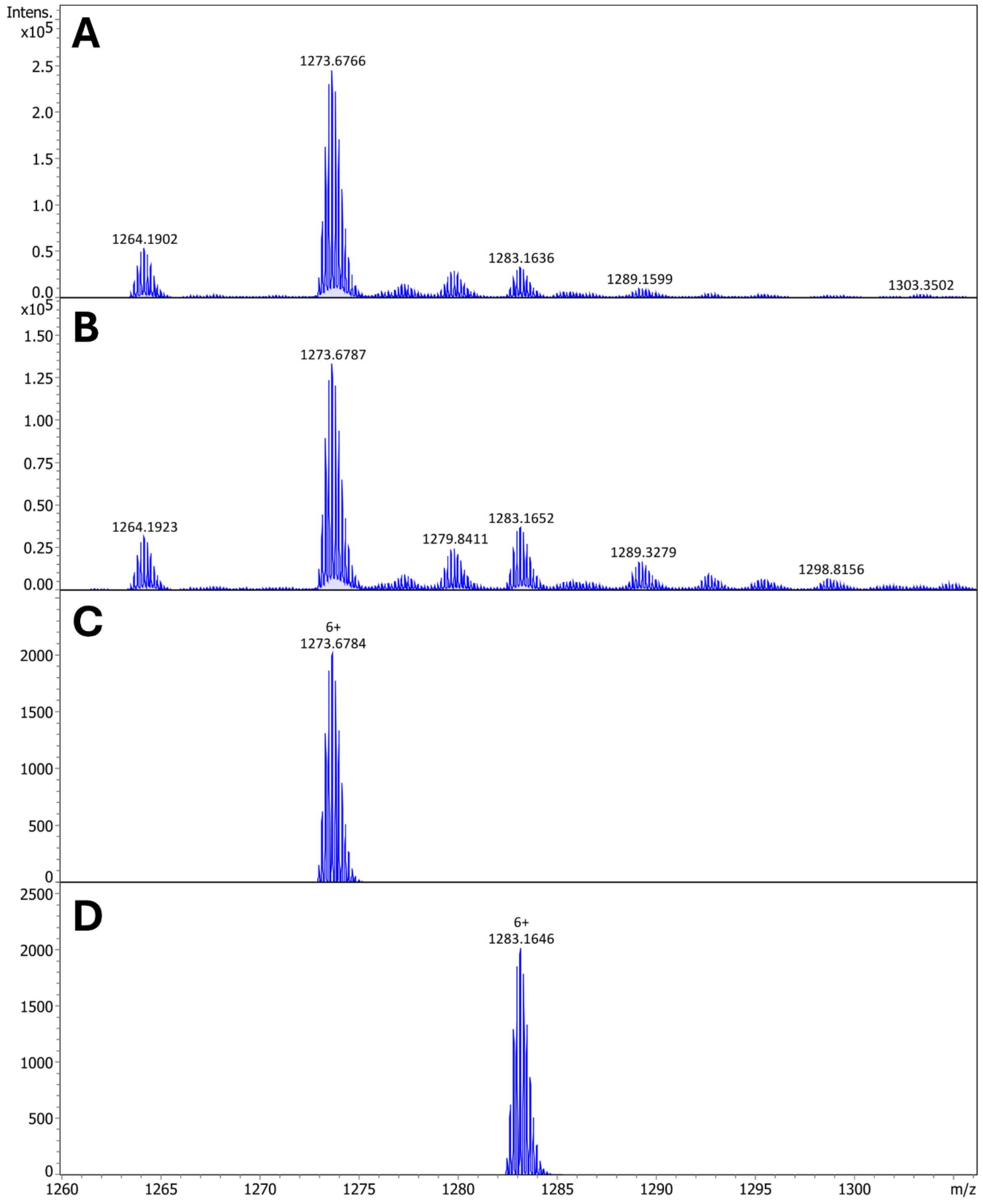

Figure S13. Native electrospray ionization mass spectrometry of 15 µM 3Hep and variable concentrations of $CoCl_2$ in 20 mM ammonium acetate. Only the 6+ peaks are shown. **A)** Measured spectrum of 3Hep – Co(II) 1:3. **B)** Measured spectrum of 3Hep – Co(II) 1:10. **C)** Simulated mass for 3Hep complexed with 1 Co(II) ion. **D)** Simulated mass for 3Hep complexed with 2 Co(II) ions.

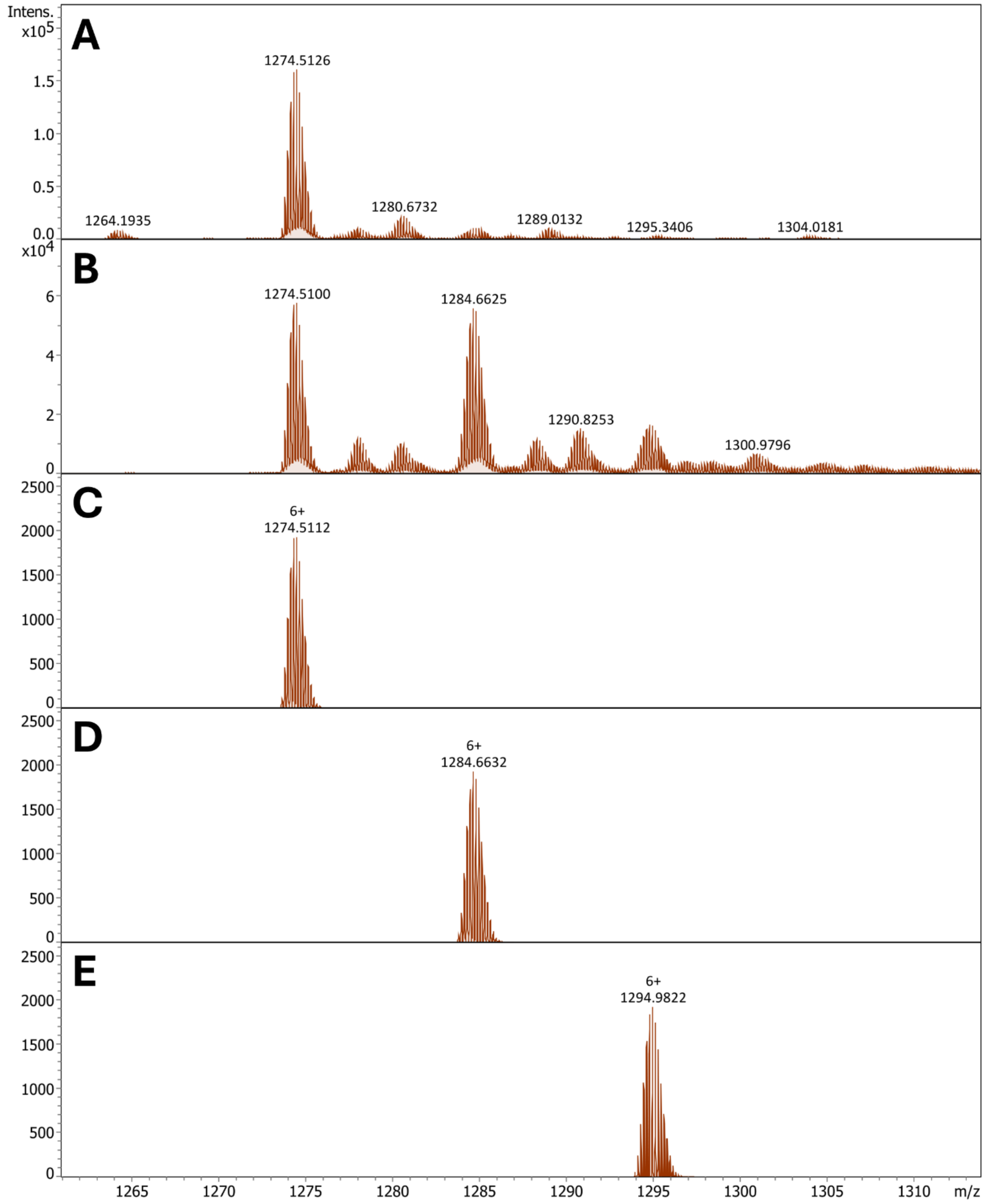

Figure S14. Native electrospray ionization mass spectrometry of 15 µM 3Hep and variable concentrations of $CuCl_2$ in 20 mM ammonium acetate. Only the 6+ peaks are shown. **A)** Measured spectrum of 3Hep – Cu(II) 1:3. **B)** Measured spectrum of 3Hep – Cu(II) 1:10. **C)** Simulated mass for 3Hep complexed with 1 Cu(II) ion. **D)** Simulated mass for 3Hep complexed with 2 Cu(II) ions. **E)** Simulated mass for 3Hep complexed with 3 Cu(II) ions.

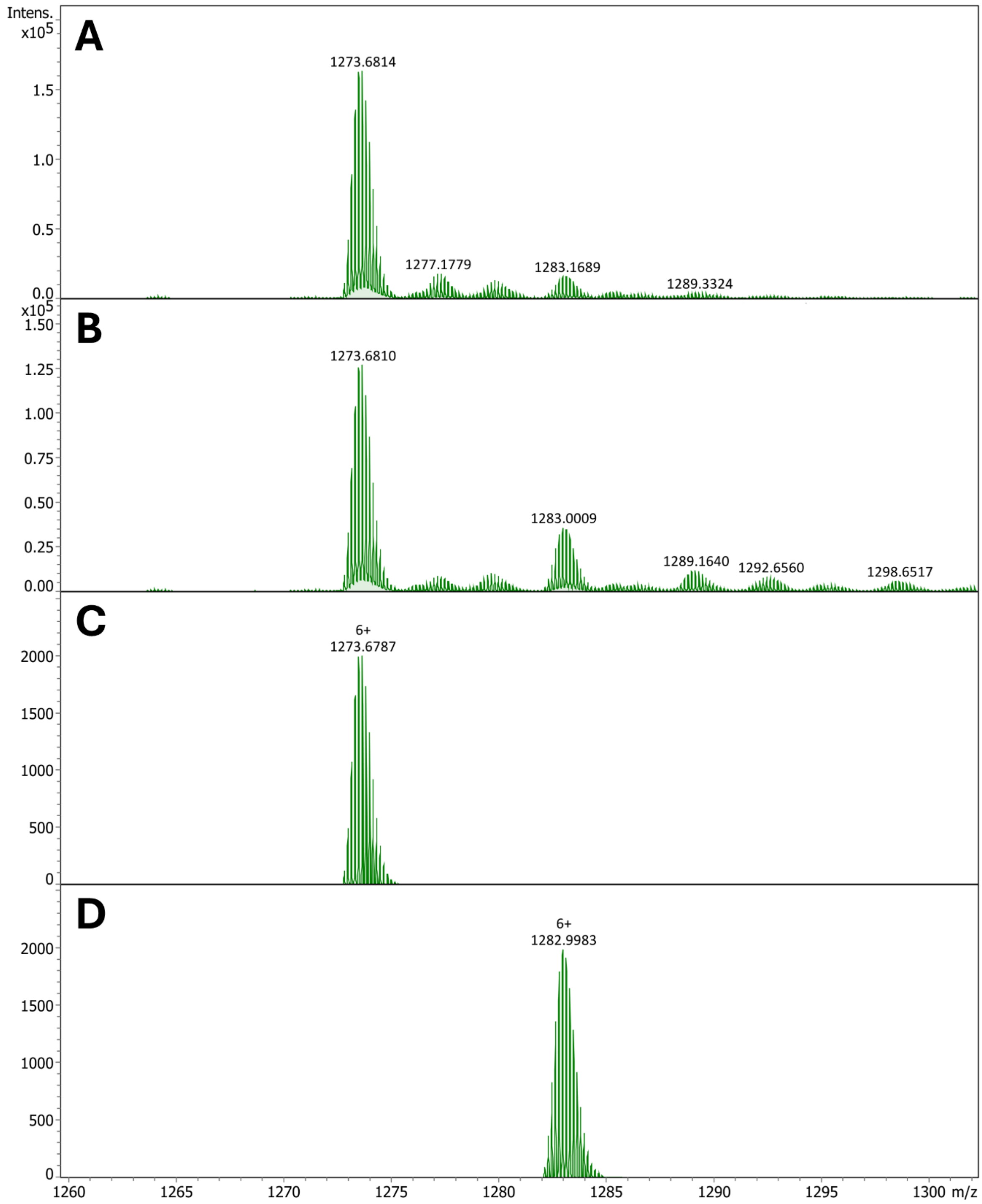

Figure S15. Native electrospray ionization mass spectrometry of 15 µM 3Hep and 150 µM $NiCl_2$ in 20 mM ammonium acetate. **A)** Measured spectrum of 3Hep – Ni(II) 1:3. Only the 6^+ peaks are shown. **B)** Measured spectrum of 3Hep – Ni(II) 1:10. Only the 6+ peaks are shown. **C)** Simulated mass for 3Hep complexed with 1 Ni(II) ion. **D)** Simulated mass for 3Hep complexed with 2 Ni(II) ions.

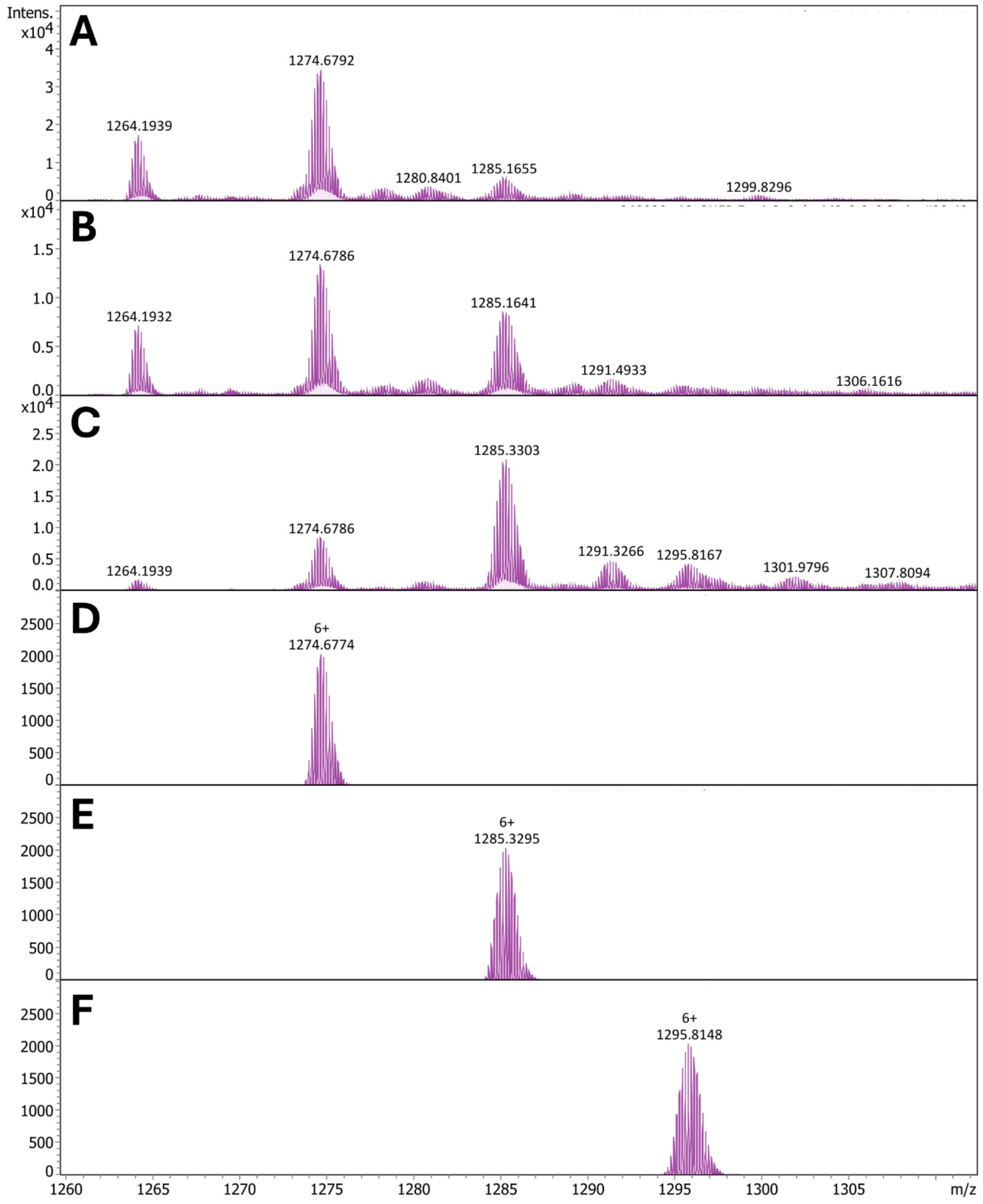

Figure S16. Native electrospray ionization mass spectrometry of 15 µM 3Hep and variable concentrations of $ZnCl_2$ in 20 mM ammonium acetate. Only the 6+ peaks are shown. **A)** Measured spectrum of 3Hep – Zn(II) 1:1. **B)** Measured spectrum of 3Hep – Zn(II) 1:3. **C)** Measured spectrum of 3Hep – Zn(II) 1:10. **D)** Simulated mass for 3Hep complexed with 1 Zn(II) ion. **E)** Simulated mass for 3Hep complexed with 2 Zn(II) ions. **F)** Simulated mass for 3Hep complexed with 3 Zn(II) ions.
